# Commemoration of Comparative Cardiac Anatomy of the Reptilia I‐IV

**DOI:** 10.1002/jmor.20964

**Published:** 2019-02-11

**Authors:** Bjarke Jensen

**Affiliations:** ^1^ Department of Medical Biology, Amsterdam Cardiovascular Sciences University of Amsterdam, Amsterdam UMC Amsterdam The Netherlands

**Keywords:** evolution, heart, *Varanus*

## Abstract

Our understanding of the anatomy of hearts of ectothermic saurosids, or colloquially “reptiles”, was much advanced by the publication of the series of four papers under the heading of Comparative Cardiac Anatomy of the Reptilia in Journal of Morphology between 1971 and 1981. Here, I commemorate the papers, show how they moved our understanding forwards, and briefly describe the state‐of‐the‐art.

Fifty years ago, in Sydney Australia, Grahame Webb finished his thesis “The squamate heart” that would become the foundation of the first two of ultimately four papers of the classical series “Comparative Cardiac Anatomy of the Reptilia” published in Journal of Morphology (MacKinnon & Heatwole, 1981; Webb, 1979; Webb, Heatwole, & Bavay, 1971; Webb, Heatwole, & de Bavay, 1974). The culmination of Webb's efforts was the authoritative and single‐authored study on the crocodylian heart from 40 years ago (Webb, 1979), while his thesis supervisor Harold Heatwole, would finish the franchise in 1981 with the authoritative study on the coronary vasculature of non‐crocodylian ectothermic sauropsid hearts (MacKinnon & Heatwole, 1981).

The first paper focused on the ventricle of the varanid lizards, or monitors, and emphasizes its prominent septa (Webb et al., 1971). Webb et al. were by no means the first to be intrigued by the monitor ventricles. Physiological experiments started by Harrison (White, 1968) and unequivocally completed by Warren Burggren and Kjell Johansen showed that the monitor ventricle is divided into a high‐pressure left side and a low‐pressure right side, much like that in mammals (Burggren & Johansen, 1982; Johansen & Burggren, 1984). Thanks to these efforts, the monitor heart is now a key case in textbooks on vertebrate anatomy and physiology to understand the evolution of the four‐chambered hearts of mammals (Synapsida) and archosaurs (Sauropsida) from the undivided heart of the ancestral amniotes (Kardong, 2006; Randall, Burggren, French, & Eckert, 2002).

Historically Brücke (Brücke, 1852) and Greil (Greil, 1903), had described the salient anatomical features of the monitor ventricle. However, like so many studies in German, these were largely forgotten, or became illegible, as spoken and written English began to dominate over German and French in scientific communication (Buchanan, 1956; Reese, 1915; White, 1959). The value of the work of Webb et al. was the clear prose and bringing together fragmented literature with inconsistent terminology “The terminology used by Harrison is extremely confusing” (Webb et al., 1974). Further, they also brought back poorly appreciated works, including the marvelous study by Greil (Greil, 1903) from which Webb and colleagues reintroduced the German terms such as Muskelleiste for the so‐called muscular ridge, or horizontal septum or folding septum, and the term Bulbuslamelle (which Brücke had given the mundane name of meat‐pillar, “Fleischpolster” [Brücke, 1852]). Indeed, Figure 9 of Webb et al. (Webb et al., 1971), which was the prime illustration of the Muskelleiste and Bulbuslamelle, is readily comparable to Figure 5 of plate VIII of Greil (Figure 1). These two septa, the Muskelleiste and the Bulbuslamelle, come together during ventricular contraction and thereby separate the pressures of the left and right side of the ventricle.

**Figure 1:**
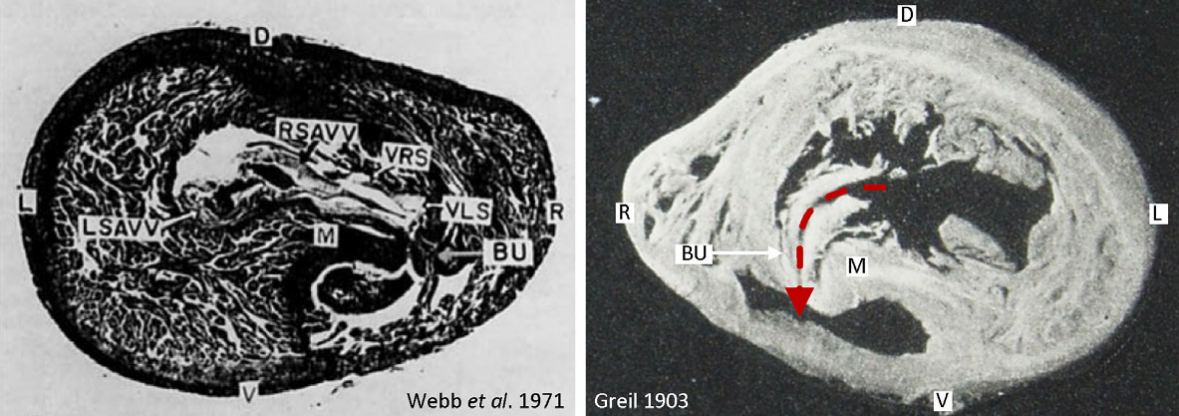
On the left, a reproduction of Figure 10 of Webb et al. (Webb et al., 1971), and on the right, Figure 5 of Tafel VIII of Greil (Greil, 1903) with complementary labeling (the image on the left is presented “as seen from behind” (Webb et al., 1971), whereas the image on the right is presented as seen from front. Webb et al. (Webb et al., 1971; Webb et al., 1974) made a very deliberate and successful attempt to bring to attention the marvelous study of Greil (Greil, 1903), including the emphasis on the two septa that divide the ventricle in systole, the Muskelleiste (M) and the Bulbuslamelle (BU). The dashed arrow on the right shows the path by which systemic venosus blood in diastole can reach the cavum pulmonale (at the arrowhead) by flowing through the narrow gap between the Muskelleiste and the Bulbuslamelle. D = dorsal; L = left; LSAVV = left septal atrio‐ventricular valve; R = right; RSAVV = right septal atrio‐ventricular valve; V = ventral; VLS = valve of the left systemic arch; VRS = valve of the right systemic arch [Color figure can be viewed at wileyonlinelibrary.com]

The deliberate attempt by Webb et al. to revive lost literature, including works in Latin, French, and Russian, also resulted in the second paper of the series (Webb et al., 1974). This article was a critique of nomenclature and although it focused on Squamata and Rhynchocephalia, its findings and recommendations also apply well to studies of hearts of Testudines (Jensen, Moorman, & Wang, 2014). The value and appreciation of reviving lost literature is acknowledged by later studies on ectothermic sauropsid hearts, also published in Journal of Morphology (Crossley & Burggren, 2009; Jensen, Abe, Andrade, Nyengaard, & Wang, 2010; Lopez et al., 2003; Starck, 2009; Young, Lillywhite, & Wassersug, 1993), as they cite Webb et al. (MacKinnon & Heatwole, 1981; Webb, 1979;Webb et al., 1971 ; Webb et al., 1974), Greil (Greil, 1903), and other classics.

In his final contribution (Webb, 1979), the findings on the crocodylian heart spurred Webb to re‐evaluate the previous papers (Webb et al., 1971; Webb et al., 1974). While his description of the crocodylian heart still stands (Cook et al., 2017), the re‐evaluations of the noncrocodylian ventricle were largely erroneous (see [Jensen et al., 2014] for a detailed discussion). Webb (Webb, 1979) emphasized correctly that it was now shown that the left side of the monitor ventricle has higher pressures than the right (Millard & Johansen, 1974), but he inferred that the monitor setting of “increased muscularization of the cavum arteriosum … applies equally to snakes,” p. 232. The python ventricle in fact resembles the monitor ventricle functionally and anatomically, although the python ventricle has a particularly reduced cavum venosum, but the ventricle of non‐python snakes is essentially an elongated variety of the typical lizard ventricle and not like the monitor ventricle [Jensen et al., 2014]. Accordingly, it is only in monitors and pythons that the pulmonary systolic blood pressure is substantially lower than the systolic blood pressure of the systemic circulation, whereas in other noncrocodylian ectothermic sauropsids the ventricle ejects blood with similar systolic pressure into the both circulations (Jensen et al., 2014). Webb (1979) also thought he had misplaced the cavum venosum in 1971 and 1974, and now suggested that the cavum venosum was the dorsal part of the cavum pulmonale, with the boundary between the two being the Muskelleiste. Moreover, what was previously thought to be the cavum venosum was now added to the cavum arteriosum and the vertical septum became the dorsal part of the Bulbuslamelle. Unfortunately, these revisions overlooked the founding observations of Brücke (1852) that we still follow (Jensen et al., 2014), namely that the cavum venosum is the chamber that (a) receives the systemic venous return in diastole and (b) is sealed by the aortic valves. When Webb was told of his erroneous revisions, because it would be discussed in an upcoming review, the clear and unceremonious prose emerged again “I … thought I'd screwed up on the original interpretation which I was keen to try and correct. Seems it created more red herrings” (personal communication, BJ, 2013). In Figure 2, I have reproduced Figure 1b of (Webb et al., 1974) which schematizes the relationship of the major septa, cavities, and valves of the noncrocodylian ventricle, which is among the most definitive overviews of existing information on these structures.

**Figure 2:**
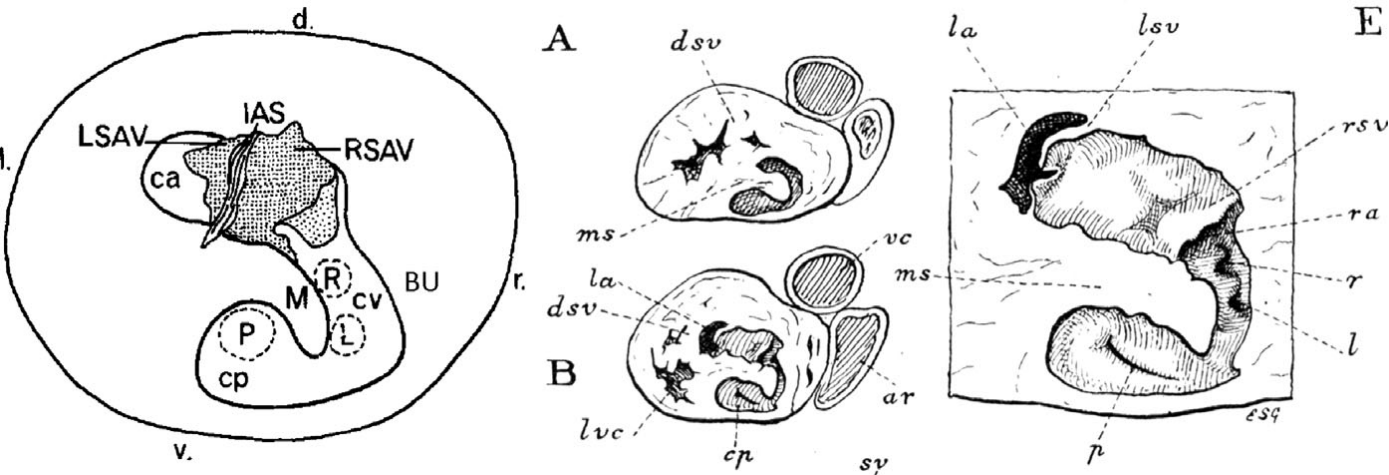
Schematic of the ventricular base of a python (*Liasis amethistinus*), showing the correct position of Muskelleiste (M), Bulbuslamelle (BU, not original label), and vertical septum (beneath the atrial septum [IAS]), which divide the ventricle into the cavum pulmonale (cp) which leads to the pulmonary artery (P), cavum venosum (cv) which leads to the left (L) and right (R) aorta, and the cavum arteriosum (ca). D = dorsal; l = left; LSAV = left leaflet of the atrioventricular valve; r = right; RSAV = right leaflet of the atrioventricular valve; v = ventral. An earlier version of this schematic appeared in Webb's thesis from 1969, which in turn appears to be based on cartoons of the heart of the Burmese python by Goodrich (Goodrich, 1919), published a 100 years ago this year and shown here on the right. dsv, muscles representing dorsal region of septum ventriculorum (vertical septum); ms, muscular interventricular incomplete septum (Muskelleiste)

Webb's 1979 paper concluded with hypotheses on the development of ventricular septation from the vertical septum and the membranous septum (and the Muskelleiste which, it was assumed, would not need to remodel much in order to contribute to the full septum; Webb, 1979). These hypotheses centered around positional changes of the vertical septum and the membranous septum. However, there are crucial developmental processes to the formation of the full ventricular septum that were unknown to Webb and, to be fair, most of the community of comparative vertebrate anatomists and physiologist: first, so‐called aortic wedging whereby the aorta moves leftward and connects to the left ventricle (rather than the membranous septum moves) and, second, the rightward expansion of the atrioventricular canal that ensures that the right atrium maintains communication with the right ventricle (rather than the muscular septum moves; Anderson, Spicer, Brown, & Mohun, 2014; Lamers, Viragh, Wessels, Moorman, & Anderson, 1995). These processes are emphasized by developmental biologists, whereas positional changes to the ventricular and membranous septa, such as suggested by Webb, are not emphasized or mentioned (Anderson et al., 2014).

Recent molecular investigations have attempted to settle the origins of the full ventricular septum of mammals (Synapsida) and archosaurs (Sauropsida). Investigations showing a gradient of the transcription factor *Tbx5* in the ventricular septum of mouse and chicken were corroborated by Koshiba‐Takeuchi et al., 2009 who reported that a smaller but similar gradient exists over the vertical septum of a turtle (*Trachemys*), but not the vertical septum of a lizard (*Anolis*). Later, Poelmann et al. (Poelmann et al., 2014) showed in another species of turtle (*Pelodiscus*) and a snake (*Pantherophis*) that the crest of the vertical septum is enriched in *Tbx5* and that there is an additional gradient of *Tbx5* on the Muskelleiste (now called the folding septum). While the newer findings questioned whether a gradient of *Tbx5* is a marker of any particular septum, Jensen et al. (Jensen et al., 2018) showed that there is indeed a gradient of *Tbx5* on the ventricular septum of the American alligator and Cuvier's dwarf caiman as expected on the basis of the initial study. The study of ventricular septum evolution is being approached by new techniques, but findings often hark back to much older findings and the last word has not been said.

In the inaugural issue of Journal of Morphology, the editor Charles Otis Whitman envisioned the journal as a “medium” to concentrate and remedy the “mixed character and scattered sources of our publications” (Whitman, 1887). Journal of Morphology has since published as many papers on ectothermic sauropsid heart morphology as The Anatomical Record and Journal of Anatomy together (as assessed from the reference list of [Jensen et al., 2014]). Indeed, the classical series of “Comparative Cardiac Anatomy of the Reptilia” brought much clarity to the “mixed character and scattered sources of our publications” and remains a key body of work to anyone interested in the anatomy and physiology of the ectothermic sauropsid heart.
